# Radioactive iodine therapy outcomes in young adults with Graves disease: a bi-center observational study

**DOI:** 10.20945/2359-4292-2025-0159

**Published:** 2025-10-07

**Authors:** Liu Xiao, Qian Tang, Yu Wang, Lin Li, Yipu Zhong, Bin Liu

**Affiliations:** 1 Department of Nuclear Medicine, West China Hospital, Sichuan University, Chengdu 610041, Sichuan Province, China.; 2 Department of Nuclear Medicine, The People’s Hospital of Jianyang City, Jianyang City 641400, Sichuan Province, China.

**Keywords:** Graves disease, Young adults, Radioactive iodine

## Abstract

**Objective:**

To evaluate the efficacy of radioactive iodine therapy and to identify determinants of treatment outcomes in young adults with Graves disease.

**Methods:**

This retrospective cohort study analyzed young adults with Graves disease who underwent radioactive iodine therapy at two tertiary medical centers in Southwest China. Patients were stratified into two groups based on therapy outcomes at 6 months post-radioactive iodine therapy: euthyroidism or hypothyroidism (therapy success) and persistent hyperthyroidism necessitating either a second radioactive iodine therapy or continuation of anti-thyroid drug therapy (therapy failure). Multivariate logistic regression and receiver operating characteristic curve analyses were employed to assess predictive factors for treatment outcome.

**Results:**

A cohort of 163 young adults with Graves disease, with a mean age of 18 years (range: 6 to 20 years) were included. The overall therapy success rate was 60.7%. Multivariate regression analysis identified that thyroid mass (OR 1.013, 95%CI 1.002 - 1.025; p-value = 0.022) and interval between diagnosis and radioactive iodine therapy (> 1 year; OR 2.471, 95%CI 1.128 - 5.415; p-value = 0.024) were risk factors associated with therapy failure. ROC curve analysis identified 38 g as the optimal thyroid mass cutoff for predicting treatment failure, demonstrating a sensitivity of 69% and specificity of 70%. The positive and negative predictive values were 60% and 78%, respectively.

**Conclusion:**

A therapy success rate of 60.7% was observed in radioactive iodine therapy in young adults with Graves disease. Larger thyroid volume and prolonged disease duration emerged as significant risk factor for therapy failure.

## INTRODUCTION

Graves disease (GD) stands as the most prevalent etiology of thyrotoxicosis in young adults (YAs) (^1,2^), accounting for approximately 10 to 15% of thyroid disorders in this population (^3^). Compared to adult patients, YAs with GD exhibit a higher frequency of disease relapse (^4^). However, the clinical management of GD in YAs poses considerable challenges for YAs, their parents and physicians, mainly due to the paucity of population-specific evidence and studies available.

Current therapeutic modalities include anti-thyroid drugs (ATDs), thyroidectomy, and radioactive iodine (RAI) therapy, each presenting distinct limitations that impact clinical decision-making (^5-7^). Although ATD therapy remains the first-line treatment for YAs with GD, its clinical utility is plagued by high recurrent rates and potential adverse effects (^8,9^). Thyroidectomy demonstrates excellent therapeutic efficacy for GD, though the procedure carries inherent risks including recurrent laryngeal nerve injury, hypoparathyroidism, and postoperative hematoma formation. Recent trends suggest a declining preference for RAI therapy as first-line treatment for YAs with GD (^10^). Nevertheless, YAs with GD who demonstrate ATD resistance, experience disease recurrence following ATD withdrawal, or develop ATD-related adverse events are typically considered candidates for RAI therapy.

The primary therapeutic objective of RAI treatment for YAs with GD is the induction of hypothyroidism, with documented effectiveness and low incidence of adverse effects compared to alternative therapy (^11,12^). Despite its clinical utility, comprehensive evaluations of RAI therapy success rates in this specific population remain limited in the current literature (^13-20^).

Our objective was to evaluate the efficacy of RAI therapy and identify determinants of treatment outcomes in young adults with GD.

## METHODS

### Patients

We conducted a bi-center retrospective study of consecutive YAs receiving RAI therapy for GD at two tertiary medical centers (West China Hospital of Sichuan University and The People’s Hospital of Jianyang City) between January 2015 and December 2023.

Patients who met the following inclusion criteria were first selected: aged 6 to 20 years at RAI treatment initiation; confirmed GD diagnosed; clinical referral for RAI therapy. Graves disease was diagnosed based on identification of suppressed thyroid stimulating hormone (TSH), positive TSH receptor antibody (TRAb), and/or diffuse high uptake of ^99m^TcO_4_- by the thyroid gland. Exclusion criteria comprised: prior RAI treatment for GD, concurrent thyroid malignancy, unavailable post-treatment biochemical follow-up data, or incomplete clinical records.

A total of 163 patients were eligible for the final analysis in this study. After appropriate institutional review board approval at each hospital, data were collected from electronic medical charts. The requirement for informed consent was waived due to the retrospective nature of the study and use of anonymized patient data, in accordance with institutional guidelines and the Declaration of Helsinki.

### Radioactive iodine therapy

Comprehensive assessments were conducted for each patient, including thyroid function tests (including TSH, free triiodothyronine [FT3], free thyroxine [FT4]). Thyroid peroxidase antibodies [TPOAb] and TRAb), RAI uptake (RAIU) tests, thyroid mass measurements, complete blood count and liver function tests. FT3, FT4, TSH, TPOAb, and TRAb levels were measured using a fully auto-mated electrochemiluminescent immunoassay analyser (Cobas®e601; Immunoassay Analyzer; Roche) with measurement ranges of zero to 50 pmol/L for FT3, zero to 100 pmol/L for FT4, 0.005 to 100 mIU/L for TSH, zero to 600 IU/mL for TPOAb, and 0.3 to 40 IU/L for TRAb, respectively. The presence of GD ophthalmopathy was confirmed by physical examination on medical records.

Radioactive iodine uptake was measured at 3- and 24-hour intervals after oral administration of a 0.37 MBq tracer dose. Thyroid gland volume was quantified ultrasonographically by experienced operators using high-resolution color Doppler systems equipped with multifrequency probes. The ellipsoid model for the thyroid gland was applied to determine the thyroid mass of each patient, where the density was considered to be approximately 1.0 g/cm^3^.

The ^131^I dose was calculated based on the following formula:

$${}^{131}\text{I treatment dose = }\frac{\text{intended dose (µCi/g)}\times \text{thyroid weight (g)}}{24-\text{hours RAIU}(\%)}$$

The intended dose ranged from 2.59-4.44 MBq/g of thyroid tissue. For patients undergoing ATD therapy, methimazole was withdrawn for a minimum of 3 days and propylthiouracil PTU for 14 days before RAI administration to minimize interference with iodine uptake.

### Clinical outcomes

Post-therapy assessment of all patients was conducted at 6 months following RAI therapy. Successful RAI therapy was defined as achieving either a biochemical euthyroid or hypothyroid state. Biochemical euthyroid was defined as normal TSH, FT3 and FT4 level. Hypothyroid was considered as elevated TSH level and normal or reduced FT3 and FT4 level.

Patients were categorized into two groups based on therapy outcomes: those achieving euthyroid or hypothyroid status (therapy success) and those experiencing persistent hyperthyroidism necessitating either a second RAI therapy or re-continuation of ATD therapy (therapy failure).

### Statistical analysis

Continuous variables were presented as mean ± standard deviation (SD) for normally distributed data, or median with interquartile range (25th-75th percentile; P25-P75) for non-normally distributed data. Categorical variables were expressed as absolute numbers and percentages. Comparative analyses utilized unpaired Student’s *t*-test for normally distributed data, Mann-Whitney U-test for non-normally distributed data, and Pearson’s chi-squared test for categorical variables. Multivariate logistic regression analysis was conducted to assess risk factors associated with therapy failure. Receiver Operating Characteristic (ROC) curves were employed to identify the optimal cutoff values for predicting clinical outcomes. Statistical analysis was performed using Statistical Package for Social Sciences (SPSS) software (version 22.0, SPSS Inc., Chicago, IL, USA), with significance set at p < 0.05.

## RESULTS

### Patient characteristics

A total of 163 YAs with GD were included in the study, comprising 120 females (73.6%), with a mean age of 18 years (range: 6 to 20 years). Among the cohort, 133 patients (91.9%) had received ATD therapy prior to RAI therapy (Table 1). The primary indications for radioiodine (RAI) therapy are presented in Table 2. The most common reasons were poor response/unable to withdraw ATD (n = 59; 36.2%), patient preference (n = 38; 23.3%), hepatic dysfunction (n = 35; 21.5%), and ATD-associated leukopenia (n = 19; 11.7%).

**Table 1:** Demographic characteristics and clinical data for young adults with Graves disease

Variable	Total patients (n = 163)	Treatment success group (n = 99)	Treatment failure group (n = 64)	p-value
Age, years, range	18 (^6-20^)	18 (^6-20^)	18 (^12-20^)	0.967
Gender				
Male	43 (25.2%)	31 (31.3%)	12 (18.8%)	0.076
Female	120 (74.5%)	68 (68.7%)	52 (81.3%)	
Interval between diagnosis and RAI treatment, year				
< 1	61 (35.8%)	44 (44.4%)	17 (22.6%)	0.021
> 1	102 (64.2%)	55 (55.6%)	47 (73.4%)	
ATD treatment				
Yes	133 (91.1%)	77 (77.8%)	56 (87.5%)	0.118
No	30 (8.9%)	22 (22.2%)	8 (12.5%)	
Methimazole dose, mg	15 (7.5-20)	15 (7.5-20)	15 (^10-20^)	0.232
Ophthalmopathy				
Yes	58 (38.2%)	32 (32.3%)	26 (40.6%)	0.28
No	105 (61.8%)	67 (67.7%)	38 (59.4%)	
TPOAb, IU/mL				
Negative	17 (11.4%)	11 (11.1%)	6 (9.4%)	0.723
Positive	146 (88.6%)	88 (88.9%)	58 (90.6%)	
3h-RAIU	0.56 ± 0.18	0.53 ± 0.19	0.61 ± 0.16	0.013
24h-RAIU	0.73 ± 0.14	0.72 ± 0.15	0.74 ± 0.13	0.495
3h-RAIU/24h-RAIU	0.77 ± 0.22	0.73 ± 0.19	0.84 ± 0.24	0.003
Thyroid mass, g	41.68 ± 23.93	34.59 ± 19.10	52.63 ± 26.54	<0.001
Treatment dose, mCi	8.7 ± 2.4	8.3 ± 2.3	9.2 ± 2.4	0.013
TSH, mIU/L, 0.27-4.2	0.005 (0.005-0.008)	0.005 (0.005-0.009)	0.005 (0.005-0.008)	0.701
FT4, pmol/L, 12-22	60.85 (33.66-100)	56.88 (29.25-100)	64.01 (34.07-100)	0.195
FT3, pmol/L, 3.6-7.5	25.9 (14-39.7)	21.64 (12.65-38.1)	26.73 (14.91-49.4)	0.107
TRAb at pre-RAI, IU/L, <1.75	16.37 (6.65-32.53)	13.89 (5.1-25.1)	20.54 (11.54-39.92)	0.003

Results expressed as mean ± standard deviation for normally distributed data, or median with interquartile range (25th-75th percentile) for non-normally distributed data. Categorical variables were expressed as absolute numbers and percentages. RAI: radioactive iodine; ATD: anti-thyroid drugs; TPOAb: thyroid peroxidase antibodies; TSH: thyroid stimulating hormone; FT4; free thyroxine; FT3: free triiodothyronine; TRAb: thyroid stimulating hormone receptor antibody.

**Table 2:** The reason for radioactive iodine treatment

Reason	Patient number
Poor response/unable to withdraw ATD	59
ATD allergy	10
Hepatic dysfunction	35
Leukopenia	19
Patient preference	38
Thyrotoxic periodic paralysis	2

ATD: anti-thyroid drugs.

### Clinical outcomes

At the 6-month follow-up after RAI therapy, 89 patients (54.6%) achieved a hypothyroid state, and 10 patients attained euthyroidism. Sixty-four patients (39.3%) remained hyperthyroid. Among treatment failures, 64.1%(41/64) underwent repeat RAI therapy, while 35.9% (23/64) resumed ATD treatment. The overall treatment success rate (achievement of either hypothyroid or euthyroid status) was 60.7% (99/163).

### Predictors of therapy failure at univariate and multivariate analyses

On univariate analysis (Table 3), the following variables had significant association with clinical outcomes: interval between diagnosis and RAI therapy, thyroid mass, 3-hour RAIU level, 3h-RAIU/24-RAIU ratio, and TRAb level at pre-RAI.

**Table 3:** Risk factors of treatment failure for radioactive iodine treatment by univariate and multivariate regression analysis

Factor	Univariate analysis		Multivariate analysis	
	OR (95%CI)	p-value	OR (95%CI)	p-value
Age*	1.049 (0.929-1.185)	0.440		
Gender, female	0.845 (0.708-1.101)	0.076		
Interval between diagnosis and RAI treatment, > 1 year	1.673 (1.053-2.659)	0.021	2.471 (1.128-5.415)	0.024
ATD treatment, yes	1.778 (0.843-3.747)	0.118		
Ophthalmopathy, yes	1.14 (0.893-1.455)	0.28		
TPOAb, yes	1.185 (0.461-3.045)	0.723		
3h-RAIU*	1.023 (1.004-1.041)	0.015	0.986 (0.954-1.108)	0.381
24h-RAIU*	1.008 (0.986-1.03)	0.493		
3h-RAIU/24h-RAIU*	1.023 (1.007-1.04)	0.005	1.015 (0.985-1.046)	0.328
Thyroid mass, g*	1.109 (1.01-1.028)	<0.001	1.013 (1.002-1.025)	0.022
Treatment dose, mci*	1.187 (1.033-1.363)	0.016	1.003 (0.831-1.21)	0.977
FT4, pmol/L*	1.006 (0.996-1.016)	0.225		
FT3, pmol/L*	1.015 (0.995-1.037)	0.148		
TRAb at pre-RAI, U/L*	1.039 (1.014-1.066)	0.003	1.025 (0.994-1.056)	0.114

OR odds ratio; 95%CI:95% of confidence interval; RAI: radioactive iodine; ATD: anti-thyroid drugs; TPOAb: thyroid peroxidase antibodies; FT4; free thyroxine; FT3: free triiodothyronine; TRAb: thyroid stimulating hormone receptor antibody.

On multivariate analysis, thyroid mass (OR 1.013, 95%CI 1.002 - 1.025; p-value = 0.022) and interval between diagnosis and RAI therapy (> 1 year; OR 2.471, 95%CI 1.128 - 5.415; p-value = 0.024) was independently predicting therapy failure.

**Figure 1:**
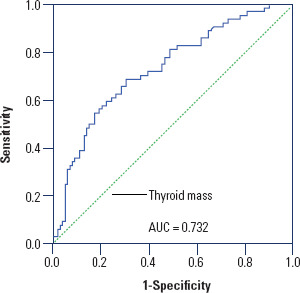
Receiver Operating Characteristic curve to determine the optimal cut-off value of thyroid mass to predict treatment failure. AUC: area under the curve.

ROC curve analysis was conducted to evaluate the predictive value of thyroid mass for RAI therapy failure. The result yielded an area under the curve (AUC) of 0.732 (95%CI 0.653-0.811; p-value < 0.001), indicating moderate predictive accuracy. The optimal thyroid mass cutoff was determined to be 38 g, demonstrating a sensitivity of 69% and specificity of 70%. This threshold showed a positive predictive value of 60% and negative predictive value of 78% (Figure 1). Notably, patients with thyroid mass >38 g had significantly lower treatment success rates (57.2%).

## DISCUSSION

In summary, the current study demonstrated a 60.7% success rate for RAI therapy in YAs with GD when using a calculated dose. Additionally, a larger thyroid mass and a longer interval between diagnosis and RAI therapy were associated with a poorer treatment response.

In our study, the therapeutic success rate of RAI therapy for YAs with GD was 60.7%. Current treatment strategies for YAs with GD are largely extrapolated from adult clinical practice, due to both the relatively low incidence of the disease in this population and the lack of prospective studies specifically assessing RAI therapy outcomes in YA cohorts. In adult GD patients, studies have reported conflicting results on factors influencing treatment outcomes, with some identifying thyroid mass (^21,22^), TRAb level (^23^), FT4 level (^22,23^), RAI dose (^21^), positive TPOAb (^24^), and prior ATD therapy (^25^) as potential factors. Limited studies have examined the factors influencing RAI therapy outcomes in YAs with GD. As summarized in Table 4, existing publications report varying efficacy rates and influencing factors for RAI therapy in this population. Notably, due to the absence of consensus on optimal dosing, the administered RAI doses varied substantially across studies, ranging from 2.96 to 14.8 MBq per gram of thyroid tissue. Overall, the success cure rate in YAs with GD after RAI therapy ranged from 42.8% to 97.5%, which was difficult to compare due to differences in the follow-up time and dose of RAI employed (^26^). The administered RAI doses in our cohort (2.59 to 4.44 MBq/g of thyroid tissue) were comparatively lower than conventional ablation doses, potentially contributing to the observed moderate treatment efficacy.

**Table 4:** previous research about treatment outcomes following radioactive iodine for young adults with Graves disease

Author	Number	Age (year)	Treatment dose	Efficacy evaluation time	Success rate	Risk factors of treatment failure
Namwongprom and cols. (^13^)	32	8-17	8.14 MBq/g	6 months	43.75% hypothyroidism 21.87% euthyroid	Lower 24h-RAIU and rapid turnover
Namwongprom and cols. (^14^)	27	7-20	5.55 MBq/g	6 months	40.7% hypothyroidism 14.8% euthyroid	Not available
Rivkees and cols. (^15^)	31	7-18	2.96-4.44, 7.4-9.25 9.99-13.32 MBq/g	1 year	Hypothyroidism: 50%, 75%, 95%	Larger thyroid
Kaplowitz and cols. (^16^)	72	6-18	4.44-14.8 MBq/g	6 months	73% hypothyroidism 4% euthyroid	Larger thyroid volume and lower treatment dose
McCormack and cols. (^17^)	48	7-20	5.92 MBq/g	6 months	73% hypothyroidism	previous ATD treatment, ophthalmopathy
Hayek and cols. (^18^)	28	8-18	74-740 MBq	1 year	17.8% hyperthyroidism	Not available
Azizi and cols. (^19^)	136	15±2	7.4 MBq/g	1 year	14.7% hyperthyroidism	Not available
Pinto and cols. (^20^)	22	12.7±4	3.7 MBq/g	6 months	73% hypothyroidism	Not available
Sheremeta and cols. (^27^)	144	8-18	744-1084 MBq	12 months	93% hypothyroidism	Larger thyroid volume

RAIU: radioactive iodine uptake; ATD: anti-thyroid drugs.

Our study identified thyroid mass (> 38 g) as a significant prognostic factor. This finding is partially supported by previous studies, which reported thyroid mass (^15,16^), 24-RAIU (^13^), previous ATD therapy (^17^), the presence of ophthalmopathy (^17^) and RAI dose (^16^) as factors influencing treatment outcomes in YAs with GD undergoing RAI therapy. These studies share common methodological limitations, including reliance on single-institution data, relatively small sample sizes, and exclusive use of univariate analyses. These findings are corroborated by Sheremeta and cols., who identified thyroid mass > 45 g as a key predictive factor for RAI therapy outcomes in their cohort of 144 patients 927. However, Sheremeta and cols. study was limited by the absence of multivariate analysis to more precisely determine the independent predictive value of thyroid mass. Larger thyroid volumes may dilute the administered RAI dose, reducing the effective radiation dose per unit volume of thyroid tissue. This can result in sub-optimal ablation of thyroid cells and higher treatment failure rates. Our findings support the clinical consideration of dose escalation for YAs with substantial thyroid volumes to improve euthyroidism achievement rates.

Our study further demonstrated that prolonged disease duration adversely affects RAI therapy response in YAs with GD, aligning with existing findings in adult populations (^28^). The chronic inflammatory milieu in longstanding GD may promote thyroid tissue remodeling through immune-mediated mechanisms, potentially reducing the radiosensitivity of follicular cells. This pathophysiological alteration could explain the diminished therapeutic efficacy observed in patients with extended disease courses. Early RAI intervention in YAs with GD may optimize therapeutic efficacy by targeting thyroid tissue prior to the development of extensive immune-mediated remodeling.

Multivariate analysis in our study revealed no significant association between RAI dosage and treatment outcomes. This finding may be explained by several considerations. First, heterogeneity in baseline patient characteristics, particularly thyroid volume and disease duration, likely confounded the dose-response relationship. For example, while patients presenting with larger thyroid volumes or prolonged disease duration may theoretically require increased RAI doses to achieve euthyroidism, these same clinical characteristics may independently confer intrinsic treatment resistance, potentially diminishing the dose-response relationship. Meanwhile, we observed a paradoxical association between treatment failure and higher administered RAI doses. This pattern likely reflects clinicians’ tendency to prescribe escalated doses for high-risk patients (e.g., those with substantial thyroid enlargement or severe disease manifestations). However, these elevated doses failed to improve therapeutic efficacy, potentially due to radiation-induced thyroid fibrosis or impaired iodine avidity in chronically inflamed tissue. Personalized dosing protocols, incorporating factors like thyroid volume, iodine uptake rates, and disease duration, may improve treatment efficacy and reduce the risk of failure.

While the two-center design of our study represents a strength that enhances the reliability and generalizability of our findings, the investigation shares limitations common to most studies involving YAs with GD. Specifically, the rarity of GD in this population inherently restricted our sample size, consequently limiting the statistical power to draw definitive conclusions. First, the recommended therapeutic dose of ^131^I in China is generally lower than that in Western countries. According to the 2016 American Thyroid Association (ATA) Guidelines for the Diagnosis and Management of Hyperthyroidism, an administered activity of at least 5.55 MBq (150 µCi) per gram of thyroid tissue is recommended to achieve hypothyroidism (^29^). Higher, individualized RAI doses may enhance treatment efficacy. Meanwhile, total thyroidectomy represents an excellent definitive treatment option, particularly in centers with experienced pediatric thyroid surgeons. Compared to RAI therapy, thyroidectomy is associated with significantly lower rates of re-treatment for GD. Nevertheless, RAI remains a valuable alternative for patients who prefer a non-surgical approach or lack access to high-volume surgical centers. Second, the follow-up duration in our study was relatively short. Future studies should incorporate extended observation periods to assess the long-term outcomes of RAI therapy in YAs with GD. Additionally, the retrospective design of this study carries inherent limitations, including potential selection bias and incomplete data collection, which may affect the robustness of our findings.

## Conclusion

A therapy success rate of 60.7% with a calculated dose for radioactive iodine therapy in young adults with Graves disease was observed. Larger thyroid volume and longer disease course emerged as significant risk factor for therapy failure. These findings highlight the need for future research to explore optimized radioactive iodine dosing protocols and treatment timing strategies tailored to these risk factors, which may enhance therapeutic outcomes in this patient population.

## Data Availability

datasets related to this article will be available upon request to the corresponding author.
